# Trends in esophageal cancer and body mass index by race and gender in the state of Michigan

**DOI:** 10.1186/1471-230X-9-47

**Published:** 2009-06-23

**Authors:** Eric J Kort, Eric Sevensma, Timothy L Fitzgerald

**Affiliations:** 1 Laboratories of Molecular Epidemiology and Cancer Genetics, Van Andel Research Institute, Grand Rapids, MI 49503, USA; 2 Ingham Regional Medical Center, Department of Surgery, Lansing, MI 48910, USA; 3 Division of Surgical Oncology, Brody School of Medicine, East Carolina University, Greenville, NC, 27834, USA

## Abstract

**Background:**

Adenocarcinoma of the esophagus has been increasing in incidence in the U.S. over the past several decades, particularly among white males. The factors driving the racial disparity in adenocarcinomas rates are not well understood.

**Methods:**

Here we examine trends in both esophageal cancer incidence and body mass index (BMI) in a geographically defined cohort by gender and race. Age-adjusted esophageal cancer incidence rates from 1985 to 2005 were calculated from data collected by the Michigan state cancer registry. Trends were analyzed along with trends in BMI data obtained from the Behavioral Risk Factor Survey administered by the Centers for Disease Control.

**Results:**

Overall, age adjusted incidence rates in esophageal carcinoma increased from 4.49 to 4.72 cases/100,000 persons per year in Michigan from 1985 to 2005. Among white males, the rate of adenocarcinomas increased by 0.21 cases/100,000 per year to a maximum of 6.40 cases/100,000 in 1999, after which these rates remained constant. There was a slight but non-significant increase in the rate of adenocarcinomas among African American males, for whom the average incidence rate was 8 times lower than that for white males (0.58 vs 4.72 cases/100,000 person years). While average BMI is rising in Michigan (from 26.68 in 1988 to 30.33 in 2005), average BMI was slightly higher among African Americans on average, and the rates of increase in BMI were not different between African American males and white males.

**Conclusion:**

The disparity between African American males and white males is not explained by ecological-level trends in BMI. Further research to identify the factors responsible for this disparity, possibly including anatomic fat distribution, are required.

## Background

Esophageal cancer has been increasing in incidence in the U.S. over the past several decades, despite the fact that incidence rates of the squamous cell subtype has been declining rapidly. This is the result of the even more rapid rise in incidence rates of adenocarcinoma of the esophagus [1-5]. The risk factors for squamous cell carcinoma are well understood; low income, smoking, high alcohol intake, and low intake of raw fruits and vegetables can account for up to 98% of cases of squamous cell carcinoma of the esophagus [6-8]. Furthermore, differences in these risk factors account for the much higher incidence of this tumor subtype among African American males compared to white males.

Less well understood are the factors underlying the rising incidence in adenocarcinomas of the esophagus, which is now by far the most common form of the disease. One important factor associated with the rise in incidence of adenocarcinomas is increased body mass index (BMI). A relationship between increased BMI and esophageal adenocarcinoma risk has been demonstrated in both cohort [9-11] and case control [12,13] studies. In addition, reflux is a well defined risk factor for esophageal adenocarcinoma [8,14], and it has traditionally been assumed that the effect of elevated BMI is etiologically mediated by reflux. However, recent evidence suggests that the relationship between BMI and reflux and esophageal adenocarcinoma is synergistic – i.e., that elevated BMI has some effects on the development of disease independent of reflux [12]. However, it remains unclear to what extent these risk factors explain the fact that the disease is most common, and is rising most rapidly, among white males compared to other groups. Furthermore, prior case control studies have typically matched on race (precluding analysis of race in multivariate risk models) and have not stratified their analyses of BMI and esophageal carcinoma risk by race.

Understanding the temporal and geographic trends in esophageal cancer, including its relationship to BMI, will promote our ability to identify the risk factors contributing to these trends. Significant geographic variation in the incidence of esophageal cancers has been noted previously [15,16]. In this report, our goal is to add to this body of evidence by reporting trends in gender- and race-stratified esophageal cancer rates as documented in the State of Michigan's tumor registry and to examine these trends in light of ecological BMI data.

## Methods

Statutory reporting of cancer cases to the state registry has been in place in the state of Michigan since 1985. For this report, we compiled public use incidence data from this database. As the data is anonymized and in the public domain, it is exempt from IRB review. The number of cases of esophageal adenocarcinomas and squamous cell carcinoma by age, gender, and race was obtained from the public use files maintained by the registry for the years 1985–2005. Esophageal adenocarcinomas was defined as ICDO codes 81403, 81443, 81903, 82103, 82113, 82553, 82603, 82613, 82623, 82633, 83103, 83233, 84103, 84803, 84813, and 84903. Squamous cell carcinoma was defined as ICDO codes 80763, 80713, 80723, 80733, 80743, 80753, and 80763.

Incidence rates per 100,000 persons per year were calculated using Michigan population estimates obtained from the U.S. Census Bureau. Rates were age adjusted by the direct method using the 2000 Michigan population as the standard.

Change over time was summarized using two metrics: average percent change and average rate of change. Average percent change was calculated as 100 * (e^β^-1) where β is the slope of the best fit regression line for the (natural) log-transformed incidence rates over time. Average rate of change was simply the slope of the best fit regression line for the non-transformed incidence rates over time.

It was our subjective impression that the rate of change in adenocarcinoma incidence shifted significantly among white males in 1999. To verify this, we performed joinpoint regression to identify the significant inflection points in this data, and analysis of covariance to confirm a statistical difference in slope before vs. after 1999.

To complement our analysis of esophageal cancer incidence rates by gender and race, we also performed an ecological analysis of body mass index using data from the CDC's Behavioral Risk Factory Survey. Michigan began participating in this survey in 1988. The BMI data from this dataset – based on self reported height and weight – provides only a crude perspective on the relationship between body mass and esophageal cancer risk; nonetheless, they represent the only retrospective state-wide sample available and we report them here to glean from them what etiologic hints we can.

## Results

The cancer registry recorded a total of 9165 individuals diagnosed with esophageal carcinoma over the 20 years studied: 4551 with squamous cell carcinoma and 4614 patients with adenocarcinoma. The overall age-adjusted incidence rates of esophageal carcinomas increased slightly over this time period, from 4.49 to 4.72 cases per 100,000 persons per year. However, the trend over time was not significant.

**Figure 1:**
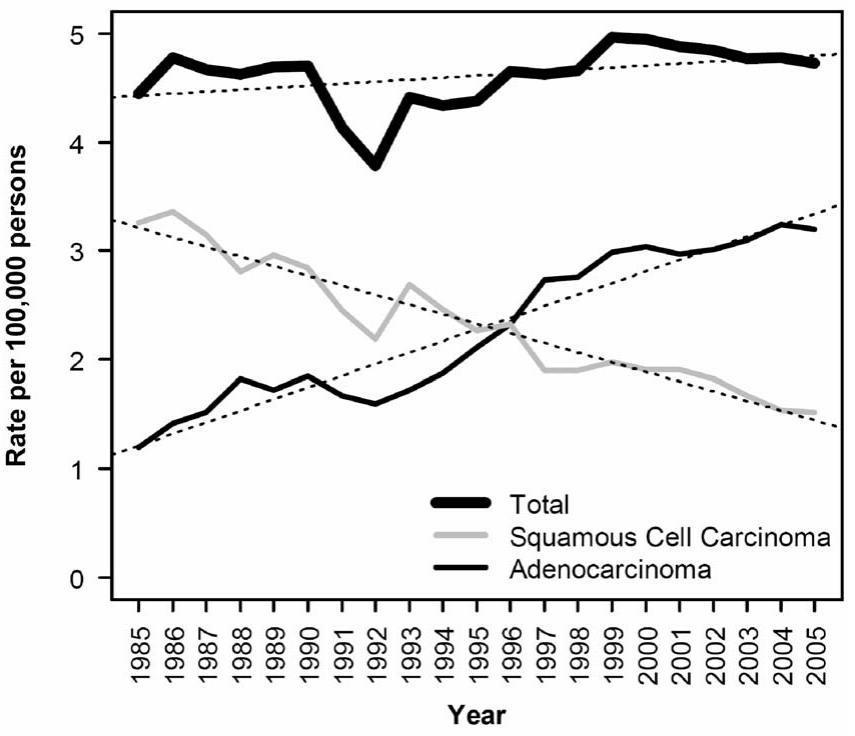
**Incidence rates of esophageal carcinoma, by year and histological subtype, for all races combined**. Dotted lines represents the best fit regression line (p < 0.001 for both trends shown). Rates have been adjusted to the 2000 Michigan population.

**Figure 2:**
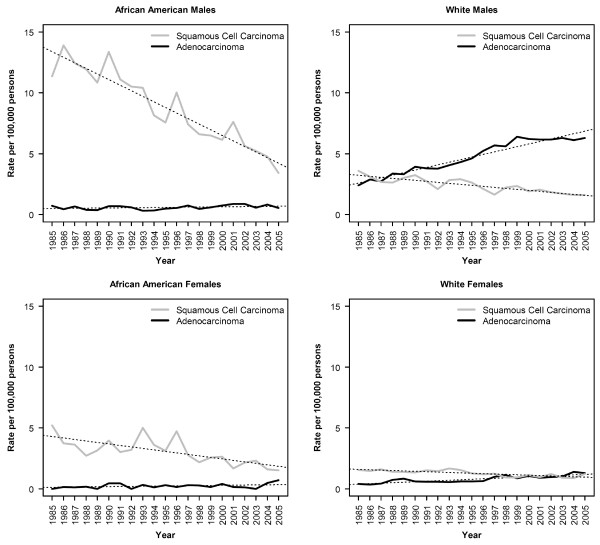
**Incidence rates of esophageal carcinoma, by year, race, gender and histological subtype, for all races combined**. Dotted lines represent the best fit regression lines (p < 0.001 for all trends shown except adenocarcinomas among African American males and females for which the trend is not significant). Rates have been adjusted to the 2000 Michigan population.

This relatively stable total incidence rates was the result of dramatic, but inverse, shifts in the incidence of adenocarcinomas and squamous cell carcinomas of the esophagus (Figure 1). Overall, the average annual change in rate of adenocarcinoma from 1985 to 2005 was 0.1065 cases per 100,000 per year, corresponding to an average percent change (APC) of 4.97% (Table 1). Squamous cell carcinoma decreased over the same period by -0.0881 cases per 100,000 per year on average, give an APC of -3.74%. In terms of average annual change in rate, squamous cell carcinoma decreased most rapidly among African American males (Figure 2a). White males and African American females (Figure 2b and 2c) had intermediate rates of decline, while white females had the lowest rate of decline (Figure 2d). As a result of these relative rates of decline, the incidence rates of esophageal squamous cell carcinoma in these four subgroups are rapidly converging. Nonetheless, African American males continue to have significantly higher incidence rates for this tumor type, albeit by a smaller margin than ever before.

**Table 1:** Rate of change in annual incidence of esophageal cancer.

		Squamous Cell Carcinoma		Adenocarcinoma	
			
Race	Gender	APC	Slope	APC	Slope
All	Both	-3.743	-0.088	4.967	0.106
	Males	-4.178	-0.135	4.813	0.179
	Female	-3.034	-0.046	5.223	0.034
					
White	Both	-3.124	-0.057	5.161	0.128
	Males: 1985–2005	-3.423	-0.082	4.992	0.215
	-- 1985–1999			5.428	0.206
	-- 1999–2005			NS	NS
	Female	-2.729	-0.035	5.529	0.041
					
Black	Both	-5.479	-0.305	2.909	0.011
	Males	-5.869	-0.504	NS	NS
	Female	-4.549	-0.130	NS	NS

Table lists Average Percent Change (APC) and slope (average annual change in rate expressed as cases per 100,000 per year) in age adjusted incidence of esophageal carcinoma by gender and race, 1985–2005. NS: non-significant trend. All other trends were statistically significant with p-values < 0.001.

In contrast, the incidence rates of esophageal adenocarcinoma has been relatively stable for African American males and females as well as for white females (Figure 2a, c, and 2d). The increase over time in the incidence rates for white women, while modest, was statistically significant; the trend over time for African American males and females was not significant. Consistent with previous reports, there was a substantial increase in age adjusted incidence rates of esophageal adenocarcinomas for white males (Fig 2b), among whom the rate of adenocarcinomas increased by 0.25 cases/100,000 per year to a maximum of 6.40 cases/100,000 in 1999. Consistent with inspection of the curve, joinpoint regression indicated that the most significant inflection point in the incidence rates among white males occurred in 1999 (p < 0.0001). Analysis of covariance indicated a highly significant difference in the slope of the trend line from 1985 to 1999 as compared to the trend line from 1999 to 2005 (p < 0.001). After 1999, the trend does not deviate significantly from 0 (p = 0.51).

**Figure 3:**
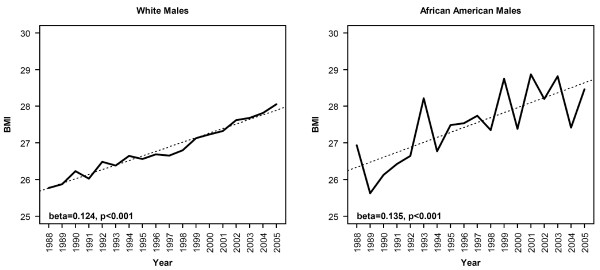
**Average BMI for Michigan males by year and race**. Dotted line represents the best fit regression lines. The slope of the best fit regression lines for African American males white males were did not differe significantly by analysis of covariance (p = 0.59).

High body mass index has been implicated as a risk factor for esophageal adenocarcinoma [8-11,17]. We investigated whether such a relationship could be supported from the data in Michigan. Though limited, our only source of a statewide sample of body mass index data for the time period under study was the CDC's behavioral risk factor survey. We extracted BMI data for Michigan respondents from this data set and compared trends in BMI to the trends observed in esophageal adenocarcinomas. As with the country as a whole, average BMI is rising in Michigan (Fig 3), from an average of 26.68 ± 11.4 (SD) in 1988 (the first year for which this data is available) to an average of 30.33 ± 14.9 (p-value for difference < 0.0001). However, there is no evidence that the rate of increase of BMI among white males is abating despite the fact that incidence of esophageal adenocarcinomas has flattened over the past 6 years in this group. The contrast between white males and African American males in terms of both incidence rates and trends in incidence rates does not appear to be attributable to differences in BMI or rate of change of BMI based on this data. Over the entire time period, average BMI was slightly higher among African American males as compared to white males (27.57 kg/m^2 ^vs. 26.90 kg/m^2^, p = 0.023). Furthermore, the rate of increase in BMI was slightly higher – though not significantly so – among African Americans (0.14 kg/m^2 ^per year vs. 0.13 kg/m^2 ^per year for whites). The opposite would be expected if rising BMI was responsible for the higher rate of increase in cancer incidence among white males.

## Discussion

Adenocarcinoma of the esophagus was a relatively uncommon malignancy at the start of the 21^st ^century and is now the most common type of esophageal cancer. Over the same period of time, there has been a decrease in the incidence of squamous cell cancers of the esophagus [1-5,18]. The etiology of this increase is unclear, but is at least is in part related to obesity and gastroesophageal reflux disease [9,19]. The preponderance of patients affected are white males [15-17,20]. Our results are similar to other reported studies in that we have found an epidemic increase in adenocarcinoma of the esophagus in white males.

Over the studied period the age-adjusted incidence rate of esophageal adenocarcinomas increased almost threefold while the incidence rate of squamous cell carcinomas decreased just over two fold. The increase in adenocarcinomas was most pronounced among white males, while the decrease in squamous cell carcinoma was most pronounced among African American males. These trends are similar to previous studies[1-5,18]. However, the trend in incidence of adenocarcinomas among white males shifted in 1999. Between 1999 and 2005, there has been no significant change in incidence of adenocarcinomas among white males in Michigan.

The etiology of the increase in adenocarcinoma of the esophagus is unclear; however, it appears to be at least partly related to obesity. Multiple studies have documented an association with BMI and esophageal adenocarcinoma including several prospective case controlled studies [8-11,17]. This has been confirmed with a metaanalysis of 2 cohort and 12 case controlled studies involving 2488 patients with adenocarcinoma esophageal cancer [14]. The relationship between obesity and adenocarcinoma of the esophagus may be related to risk of gastroesophageal reflux disease and Barrett's esophagus. Obese patients are 2.5 times more likely to have gastroesophageal reflux disease or esophageal erosions [19]. However, it has been suggested that elevated BMI may be etiologically related to esophageal carcinoma through mechanisms independent of GERD [12]. Other studies indicate that central [21] or, more specifically, visceral [19] adiposity may be more relevant than overall BMI with respect to the pathogenesis of Barrett's esophagus and subsequent cancer. Finally, it has been pointed out that GERD and Barrett's esophagus may not be the only risk factors for adenocarcinoma of the esophagus in light of the very low rate of progression from GERD to Barrett's and Barrett's to carcinoma [21].

Jeon *et al*. have previously used BMI data derived from the BRFS and noted, as we did, a rise in BMI among white Americans paralleling the rise in incidence (derived from SEER data) of esophageal adenocarcinoma [22]. This study did not report data on African Americans. We are aware of no previous report describing historical trends in BMI and esophageal cancer comparing the experience of African Americans and caucasians. While BMI has been clearly shown to be a risk factor for esophageal adenocarcinomas, it is noteworthy that African American males and white males in Michigan had similar BMIs as reported in the BRFS, and nearly identical increases in BMI over time. This analysis has several shortcomings, including the self-reported nature of the data, the sparseness of data for African Americans as indicated by the noise in the data, and ecological nature of the analysis. Nevertheless, this data demonstrates the expected rise in BMI over time, and these trends in the data are highly significant. Yet they yield no evidence that the disparity between adenocarcinoma incidence in African American males and white males can be attributed to differences in BMI. Furthermore, other risk factors (smoking, alcohol, low fruit and vegetable intake) remain somewhat more prevalent among African American males as indicated by their higher rates of squamous cell carcinoma (though these factors are rapidly improving). Finally, there was no evidence in the BRFS data that the shift in incidence trend that occurred among white males beginning in 1999 was preceded by a shift in BMI in this population.

It has been shown in previous studies that BMI-related cancer risk depends in part on the distribution of fat. There are clear gender and racial differences in the anatomic distribution of visceral fat. Whites have a high proportion of abdominal visceral fat in compared to African Americans [23,24]. Men have been found to have greater visceral adipose tissue than women [23-25]. White men, but not white women have more visceral adipose tissue than African American men and women, and their maximum visceral adipose tissue occurred within the abdomen [25,26]. There are several roles that visceral adipose tissue, particularly abdominal visceral adipose tissue, may play in etiology of adenocarcinoma of the esophagus. In obese patients, this pattern of obesity is related to Barrett's esophagus [19]. Explanations for this relationship include mechanical pressure resulting in reflux. This may also be partly related to the fact that visceral adipose tissue is hormonally active and associated with metabolic derangements [27].

## Conclusion

The incidence rate of esophageal adenocarcinoma has increased in Michigan between 1985 and 2005. This increase ceased in 1999 for white males. African Americans continue to demonstrate a rapid decline in the incidence of squamous cell carcinoma, as do the other groups examined – albeit to a lesser extent. Although mean BMI increased throughout the study, the pattern of change does not explain why adenocarcinoma is more common and was increasing more rapidly during most of the observed period among white males as compared to African American males. These findings suggest that factors in addition to BMI are responsible for the differences in incidence rates between African American males and white males. These factors may include fat distribution and/or other risk factors yet to be identified.

## Abbreviations

BRFS: Behavioral Risk Factor Survey; BMI: Body Mass Index; GERD: Gastroesophageal Reflux Disease; SEER: Surveillance, Epidemiology, and End Results program;

## Competing interests

The authors declare that they have no competing interests.

## Authors' contributions

EJK performed the analyses and drafted the manuscript. ES performed the literature review and assisted with drafting the manuscript. TF conceived of the project, guided the analysis, and assisted with drafting and reviewing the manuscript. All authors have read and approved the final manuscript.

## Pre-publication history

The pre-publication history for this paper can be accessed here:

http://www.biomedcentral.com/1471-230X/9/47/prepub
